# Preoperative Eating Patterns and Their Effect on Post-operative Outcomes in Metabolic and Bariatric Surgery: A Cohort Study of 1550 Patients

**DOI:** 10.1007/s11695-025-08094-y

**Published:** 2025-08-07

**Authors:** Adisa Poljo, Annika Wangnick, Jakob J. Reichl, Romano Schneider, Meret Appel, Amanda S. Dirnberger, Adrian T. Billeter, Marko Kraljević, Jennifer M. Klasen, Ralph Peterli

**Affiliations:** 1 Department of Visceral Surgery, Clarunis, University Digestive Health Care Center Basel, St. Clara Hospital and University Hospital Basel, Basel, Switzerland; 2 Department of Cardiology and Cardiovascular Research Institute Basel (CRIB), University Hospital Basel, University of Basel, Basel, Switzerland; 3 Department of Clinical Research, University of Basel, Basel, Switzerland

**Keywords:** Metabolic and bariatric surgery, Eating patterns, Eating disorder, Binge eating disorder

## Abstract

**Background:**

Postoperative outcomes after metabolic and bariatric (MBS) can vary due to preoperative behaviors and psychological factors, with several eating patterns (EPs), including eating disorders (EDs), often associated with suboptimal weight loss. Some guidelines even consider EDs a contraindication for MBS. This study examined the impact of EPs on long-term outcomes after MBS.

**Methods:**

A retrospective analysis was conducted on prospectively collected data from 1550 patients who underwent primary Roux-en-Y gastric bypass (RYGB) or sleeve gastrectomy (SG) between 2010 and 2018. Patients were categorized based on preoperative EPs, including binge eating disorder (BED), snacking behavior (SB), sweet eating habit (SEH), fat eating habit (FEH), night eating syndrome (NES), excessive eating habit (EEH), or no present EP. Demographics, morbidity, weight loss, comorbidities, and complications were assessed over 5 years. Outcomes were measured using the SF-BARI score.

**Results:**

EPs were common (67.6%), with many patients exhibiting multiple patterns. Patients with EPs were younger (40.8 vs. 43.3 years; *p* < 0.01) and had higher baseline BMI (44.5 vs. 43.8 kg/m^2^; *p* = 0.046) compared to patients with no EP. They showed slightly higher % total weight loss (%TWL) in the first year (31.9% vs. 30.6%; *p* < 0.01), but differences diminished over time. At 5 years, SF-BARI scores remained slightly higher in the EP group (84.2 vs. 79.1; *p* = 0.046). RYGB was performed more frequently (69.9%) and yielded better outcomes than SG.

**Conclusion:**

Preoperative EPs, including EDs, do not significantly impact postoperative outcomes after MBS, suggesting a need to reassess guidelines on EDs as contraindications

**Supplementary Information:**

The online version contains supplementary material available at 10.1007/s11695-025-08094-y.

## Introduction

Obesity remains a major public health challenge, with metabolic and bariatric surgery (MBS) standing out as the most effective intervention for long-term weight loss and improvement in obesity-related comorbidities, such as type 2 diabetes (T2D), hypertension, dyslipidemia, or obstructive sleep apnea syndrome (OSAS) [1, 2]. Although Roux-en-Y gastric bypass (RYGB) and sleeve gastrectomy (SG) are effective surgical techniques, postoperative outcomes can vary widely due to factors such as preoperative eating patterns (EPs) and psychological characteristics [3]. Investigating EPs, such as meal patterns and disordered eating behaviors, is crucial for understanding the factors contributing to severe obesity. This assessment helps identify behaviors that may need to be addressed prior to surgery. While EPs include a range of typical and non-normative behaviors that may affect health, eating disorders (EDs), as a subset of EPs, are clinically diagnosed conditions marked by severe disturbances in eating behavior and psychological distress [4, 5]. The national guidelines of the Swiss Multidisciplinary Obesity Society (SMOB) currently classify severe EDs, such as binge eating disorder (BED), as psychological contraindications for surgical therapy [6]. The American Society for Metabolic and Bariatric Surgery (ASMBS) also emphasizes the importance of evaluating EDs prior to MBS due to their potential impact on postoperative outcomes [7, 8]. BED is one of the most prevalent EDs in the general population [9]. Many patients who struggle with binge eating or loss of control eating following MBS often have a history of BED before the procedure. While estimates of BED prevalence in MBS patients vary due to differences in assessment methods, they are generally much higher compared to the general population [10, 11]. Those who develop these issues after surgery typically experience slower weight loss or greater recurrent weight gain [12]. As a result, it's essential to identify these behaviors in patients before surgery. Current data on the impact of EPs, including EDs, on postoperative outcomes following MBS remains inconsistent. Some studies suggest that preoperative EDs may negatively impact postoperative outcomes [13, 14], while other research contradicts these findings, showing no significant effect on postoperative results [15, 16]. This lack of consensus emphasizes the need for further investigation to better understand the relationship between EPs, including EDs, and MBS outcomes.

Therefore, this study aimed to investigate how various preoperative EPs, including EDs, relate to postoperative outcomes following RYGB or SG, compared to having no reported EP, in a large patient cohort.

## Material and Methods

The study was approved by the Ethics Committee of Northwestern Switzerland (reference number: 2018/00356). Data, including demographic details, early morbidity records, and follow-up information on weight loss, comorbidities, and complications, were prospectively collected for all bariatric patients who underwent surgery at Clarunis, University Digestive Health Care Center Basel. Informed consent was obtained from all participants as part of the hospital’s mandatory quality control procedures.

### Patients

In a retrospective investigation of prospectively collected data, we analyzed all patients who underwent either primary RYGB or SG in our department from January 2010 to December 2018. Patients undergoing gastric banding as their primary procedure, those undergoing revisional operations or conversion procedures were excluded, leaving 1550 patients for further analysis. In accordance with Swiss guidelines, we included individuals with a BMI of 35 kg/m^2^ or higher who had not responded to conservative treatments for at least 2 years.

Patients who met the eligibility criteria for MBS underwent a comprehensive preoperative evaluation by an interdisciplinary and interprofessional team, including specialists in medicine, nutrition, endocrinology, surgery, and psychiatry. The evaluation involved multiple appointments, educational sessions, lab tests, and personalized interventions. Post-surgery, patients were followed up by the nutrition center at least four times in the first year, twice a year from years two to five, and annually thereafter, with more frequent visits if needed. Severe and poorly controlled EPs detected by the psychiatrist were a contraindication for MBS.

### Eating Patterns and Behaviors

By taking a thorough dietary history, which included a self-reported patient diary and interviews with a specially trained dietician, and a psychiatrist, several EPs and EDs were identified. BED was diagnosed by a psychiatrist based on established criteria in the Diagnostic and Statistical Manual of Mental Disorders, 5th Edition [17, 18]. Patients were classified as having BED if they reported currently experiencing (e.g., within the past 6 months) episodes characterized by consuming an unusually large amount of food in a short period of time compared to others in similar circumstances, accompanied by a sense of loss of control during the episode.

In addition to BED, we categorized five types of eating behaviors:

- Snacking behavior (SB): defined as frequently consuming snack meals that are outside of main meals [19, 20].
- Sweet eating habit (SEH): when at least 50% of daily consumed carbohydrates consist of simple carbohydrates and which can be triggered by emotional factors (i.e., stress) [21].
- Fat eating habit (FEH): characterized by the consumption of a disproportionately high amount of fatty foods, where fats make up a significant percentage of daily calorie intake [22].
- Night eating syndrome (NES): defined by the tendency to consume large amounts of food during the night, often after the last meal of the day [23].
- Excessive eating habit (EEH): marked by an excessive intake of food during meals, significantly larger than typical portion sizes, often driven by an inability to recognize or respond to satiety signals [24].

In this manuscript, “EPs” refer to specific behavioral eating habits, which may or may not correspond to formal psychiatric diagnoses. Therefore, all of the eating behaviors described above are categorized as EPs, but only BED and NES are formally recognized psychiatric conditions characterized by pathological eating behaviors and are thus also classified as EDs.

To enhance clarity, we will from this point group the terms EDs like BED and NES and EPs like SEH collectively under the term EPs as SB, FEH, and EEH are partly included in every EP and are highly prone to subjective assessment, we excluded them from this categorization. Patients classified in the “no eating pattern” group did not exhibit any of the characteristic behaviors associated with any of the six defined EPs.

### Outcomes

The outcomes were evaluated using the SF-BARI score, which combines three components: weight loss, improvement in obesity-related comorbidities, and surgical complications, and were assessed over the first five postoperative years and compared between groups [25]. The SF-BARI score ranges from − 100 to 200 with the lowest possible value corresponding to death of the patient and further categorizing five outcome assessments from suboptimal to excellent (suboptimal < 35, fair 35 to < 70, good 70 to < 110, very good 110 to < 135, excellent ≥ 135).

The SF-BARI score has already been validated using a large external cohort of 21,605 patients from the Dutch Audit for Treatment of Obesity (DATO) and the Scandinavian Obesity Surgery Registry (SOReg) [26]. This validation demonstrated a comparable distribution of the SF-BARI score within the external cohort, supporting the generalizability of the score. Therefore, the RCT-based SF-BARI score is applicable to real-world clinical data. Furthermore, as the score is only minimally influenced by baseline characteristics, it can be reliably applied across a broad patient population.

1. Weight Loss

Total body weight loss (%TWL) was calculated using the following formula: ([weight at baseline − weight at follow-up]/weight at baseline) × 100.

Additionally excess body mass index loss percentage (%EBMIL) was calculated using the following formula: 100 − [(follow-up BMI–25/BMI at surgery–25)] × 100.

2. Comorbidities

The SF-BARI score evaluates remission of four key obesity-related conditions:

- Type 2 diabetes: remission is defined as no medication and HbA1c below 6.5%.
- Hypertension: remission is marked by discontinuation of antihypertensive medication.
- Dyslipidemia: remission is defined by an LDL-C level below 115.8 mg/dL without medication.
- Obstructive sleep apnea syndrome (OSAS): remission is based on cessation of CPAP therapy.

3. Complications

The comprehensive complication index (CCI) is used to track surgical complications defined by Clavien-Dindo > II, reflecting the individual burden of complications, with scores ranging from 0 (no complications) to 100 (death) [27].

Patients were considered “followed up” for %TWL if weight data were available at the respec-tive time point. In contrast, “follow-up” for the SF-BARI score required complete data on all five components necessary for its calculation.

### Statistical Analysis

Mean and standard deviation were used to summarize continuous data, while counts and percentages were used for categorical variables. Normality was examined using the Kolmogorov–Smirnov tests. Characteristics between the groups were compared using Fisher exact test for categorical variables and Mann–Whitney *U* test for nonparametric variables. Differences in ordinal scaled data were assessed using Pearson’s chi-squared or Kruskal–Wallis test. A *p*-value less than 0.05 was considered statistically significant. All statistical analyses were performed using R 4.2.3 (R Foundation for Statistical Computing, Vienna, Austria).

## Results

### Follow-up

Follow-up data for %TWL at 1, 2, 3, 4, and 5 years were available for 1505 (97.1%), 1446 (93.3%), 1345 (86.8%), 1280 (82.6%), and 1178 (76.0%) patients, respectively. Additionally, the SF-BARI score was available for 1147 (74.0%), 1156 (74.6%), 738 (47.6%), 555 (35.8%), and 543 (35.0%) patients at these time points.

### All Eating Patterns

Among the 1550 patients, 1048 (67.6%) were diagnosed with at least one EP. 617 patients were diagnosed with EEH, 336 with BED, 402 with FEH, 400 with SEH, 447 with SB, and 191 with NES (Table 1). Notably, most patients exhibited more than one EP, while 502 patients did not show any signs of an EP. There was a notable difference in sex distribution. The highest proportion of females was found in the SEH group (78.5%), while the lowest was in the FEH group (63.7%, *p* < 0.01). The average age across all groups was highest in patients with no EP with 43.2 ± 12.7 years (*p* < 0.01). Although patients with SEH more frequently underwent RYGB (72.0%) compared to NES (63.8%), this difference was not statistically significant. BMI, %TWL, %EBMIL, and the SF-BARI score showed no significant differences between the groups at all time points up to 5 years.

**Table 1:** Baseline-characteristics and outcomes of all eating patterns

Characteristics	Excessive eating habit (*n* = 617)	Binge eating disorder (*n* = 336)	Fat eating habit (*n* = 402)	Sweet eating habit (*n* = 400)	Snacking behavior (*n* = 447)	Night eating syndrome (*n* = 191)	No eating pattern (*n* = 502)	*p*-value*
**Sex (f)**	399 (64.6%)	257 (67.5%)	256 (63.7%)	314 (78.5%)	345 (77.2%)	123 (64.4%)	367 (73.1%)	** < 0.01**
**Age at surgery (years)**	40.9 ± 12.1	40.6 ± 12.3	41.7 ± 12.2	39.4 ± 11.5	40.7 ± 12.2	42.7 ± 12.2	43.2 ± 12.7	** < 0.01**
**Type of surgery** - RYGB (*n*) - SG (*n*)	409 (66.3%) 208 (33.7%)	228 (67.8%) 108 (32.2%)	287 (69.2%) 124 (30.8%)	288 (72.0%) 112 (28.0%)	321 (71.8%) 126 (28.2%)	122 (63.8%) 69 (36.2%)	356 (70.9%) 146 (29.1%)	0.18
**BMI (kg/m**^**2**^**)** - at baseline - 1 year - 2 years - 3 years - 4 years - 5 years	43.8 ± 6.9 30.4 ± 5.1 30.3 ± 5.2 30.9 ± 5.4 31.3 ± 5.6 31.5 ± 5.9	43.5 ± 6.6 30.7 ± 5.4 30.7 ± 5.9 31.4 ± 6.2 31.5 ± 6.1 31.8 ± 6.1	43.7 ± 6.7 30.7 ± 5.0 30.3 ± 5.2 31.2 ± 5.4 32.0 ± 5.9 32.2 ± 6.1	43.9 ± 6.9 29.9 ± 4.9 29.5 ± 5.2 30.3 ± 5.5 30.9 ± 5.9 30.5 ± 5.7	42.9 ± 6.5 30.1 ± 4.9 29.7 ± 5.2 30.5 ± 5.3 31.3 ± 5.7 31.1 ± 5.5	43.4 ± 7.6 3 0.6 ± 5.8 30.2 ± 5.7 30.7 ± 5.9 30.3 ± 4.9 31.4 ± 5.7	42.4 ± 7.1 30.1 ± 5.3 29.9 ± 5.5 30.9 ± 6.0 31.8 ± 6.0 31.9 ± 6.1	0.10 0.24 0.14 0.48 0.34 0.15
**%TWL** - 1 year - 2 years - 3 years - 4 years - 5 years	31.8 ± 7.6 31.9 ± 9.0 30.4 ± 9.6 29.4 ± 10.3 28.9 ± 10.6	31.7 ± 7.7 31.6 ± 9.5 30.0 ± 10.0 29.1 ± 10.3 27.9 ± 10.4	31.3 ± 7.6 31.9 ± 9.2 30.0 ± 9.9 28.6 ± 10.5 27.6 ± 10.6	32.2 ± 7.8 32.9 ± 9.3 31.1 ± 9.4 29.6 ± 10.2 29.4 ± 10.4	31.8 ± 8.2 32.2 ± 9.6 30.6 ± 10.2 28.3 ± 10.2 28.0 ± 10.2	31.5 ± 7.7 31.4 ± 9.1 29.8 ± 10.4 29.1 ± 10.1 28.5 ± 9.9	30.6 ± 7.2 31.5 ± 8.4 29.4 ± 9.4 28.1 ± 8.6 27.5 ± 9.3	0.10 0.52 0.62 0.79 0.57
**%EBMIL** - 1 year - 2 years - 3 years - 4 years - 5 years	75.6 ± 20.6 75.4 ± 22.6 71.9 ± 23.4 69.3 ± 24.1 68.6 ± 25.8	75.2 ± 21.9 74.6 ± 25.2 70.9 ± 26.0 69.4 ± 26.3 66.9 ± 26.4	73.8 ± 20.4 75.2 ± 22.9 70.1 ± 23.5 66.7 ± 25.3 65.1 ± 25.8	77.5 ± 20.8 78.8 ± 23.5 74.9 ± 24.0 71.2 ± 24.9 71.9 ± 25.6	76.4 ± 21.8 77.3 ± 24.2 72.9 ± 25.2 68.4 ± 24.9 68.4 ± 25.4	76.0 ± 22.5 76.2 ± 24.1 72.9 ± 26.6 72.1 ± 24.7 68.8 ± 24.6	76.3 ± 22.8 77.6 ± 24.4 71.9 ± 26.0 68.3 ± 24.4 66.7 ± 24.9	0.38 0.23 0.60 0.60 0.24
**SF-BARI score** - 1 year - 2 years - 3 years - 4 years - 5 years	89.5 ± 19.9 91.0 ± 21.7 86.9 ± 23.9 84.5 ± 25.4 84.3 ± 25.4	89.1 ± 20.5 90.9 ± 22.9 85.0 ± 24.2 83.3 ± 24.3 82.3 ± 24.9	88.1 ± 19.6 91.1 ± 22.3 84.9 ± 24.5 83.1 ± 28.0 81.5 ± 25.5	89.1 ± 20.8 92.7 ± 21.9 87.3 ± 23.2 83.2 ± 25.3 85.7 ± 25.7	88.6 ± 22.1 91.5 ± 24.1 87.7 ± 25.9 83.1 ± 25.5 84.1 ± 24.7	87.4 ± 20.7 89.3 ± 22.5 86.6 ± 26.7 83.4 ± 25.8 81.4 ± 21.9	86.5 ± 20.9 90.7 ± 22.7 82.7 ± 23.7 81.2 ± 21.9 79.1 ± 23.7	0.57 0.83 0.47 0.95 0.29

*RYGB* Roux-en-Y gastric bypass, *SG* sleeve gastrectomy, *BMI* body mass index, *%TWL* total body weight loss, *%EBMI* excess BMI loss

Values are expressed as means ± standard deviation

^*^The *p*-value represents the comparison of all six groups using appropriate statistical tests based on variable type

### Eating Pattern vs. No Eating Pattern

**Fig. 1:**
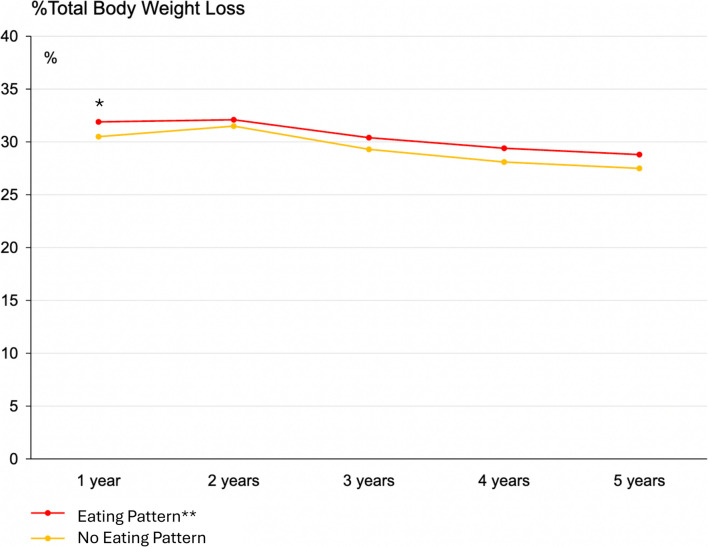
%Total body weight loss over time in patients with and without a diagnosed eating pattern; values are presented as median. *p < 0.01; binge eating disorder, sweet eating habit and night eating syndrome are summarized as “eating pattern”

**Fig. 2:**
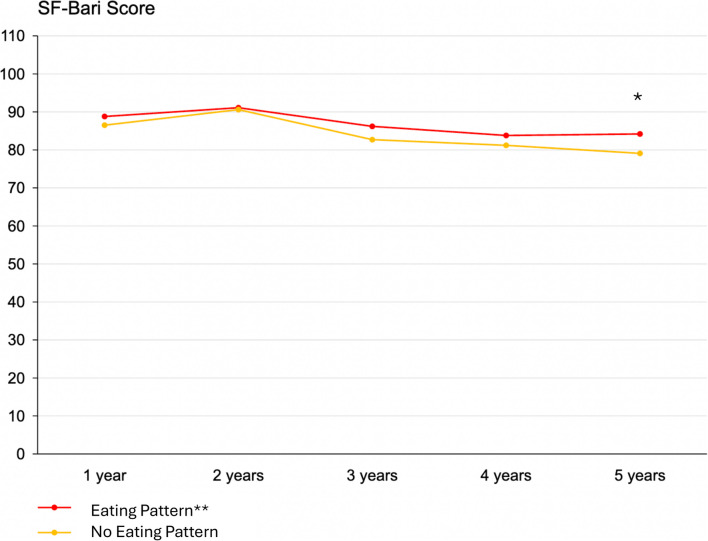
SF-BARI score over time in patients with and without a diagnosed eating pattern; values are presented as median. *p < 0.05; binge eating disorder, sweet eating habit and night eating syndrome are summarized as “eating pattern”

Patients with an EP were younger (40.8 ± 12.0 vs. 43.3 ± 12.7 years; *p* < 0.01) and had a higher baseline BMI (44.5 ± 6.6 kg/m^2^ vs. 43.8 ± 6.5 kg/m^2^; *p* = 0.046). %TWL was significantly higher in the EP group at 1 year (31.9 ± 7.8% vs. 30.6 ± 7.2%; *p* < 0.01), but this significance diminished at subsequent time points (Fig. 1). There were no differences in %EBMIL. The SF-BARI score was higher in patients with EP, but it was only statistically significant at the 5-year follow-up (84.2 ± 24.9 vs. 79.1 ± 23.7; *p* = 0.046) (Fig. 2). The results are shown in Table 2*.*

**Table 2:** Characteristics and outcomes of eating patterns vs. no eating patterns

Characteristics	Eating pattern* (*n* = 670)	No eating pattern (*n* = 502)	*p*-value
**Sex (f) (*n*; %)**	505 (75.4)	374 (74.9)	0.92
**Age at baseline (years)**	40.8 ± 12.0	43.3 ± 12.7	** < 0.01**
**Type of surgery** - RYGB (*n*; %) - SG (*n*; %)	463 (69.1) 207 (30.9)	353 (70.7) 146 (29.3)	0.59
**BMI (kg/m**^**2**^**)** - at baseline - 1 year - 2 years - 3 years - 4 years - 5 years	44.5 ± 6.6 30.3 ± 5.4 30.2 ± 5.7 30.9 ± 5.9 31.2 ± 5.9 31.2 ± 6.0	43.8 ± 6.5 30.1 ± 5.4 30.0 ± 5.6 30.9 ± 6.0 31.7 ± 6.0 32.0 ± 6.1	**0.046** 0.60 0.55 0.97 0.33 0.26
**%TWL** - 1 year - 2 years - 3 years - 4 years - 5 years	31.9 ± 7.8 32.1 ± 9.4 30.4 ± 9.8 29.4 ± 10.1 28.8 ± 10.2	30.6 ± 7.2 31.5 ± 8.4 29.4 ± 9.4 28.1 ± 8.6 27.5 ± 9.3	** < 0.01** 0.38 0.21 0.19 0.23
**%EBMIL** - 1 year - 2 years - 3 years - 4 years - 5 years	76.3 ± 21.8 76.5 ± 24.5 72.4 ± 24.9 70.3 ± 25.1 69.5 ± 25.9	76.2 ± 22.9 77.5 ± 24.4 71.9 ± 26.0 68.3 ± 24.4 66.7 ± 24.9	0.97 0.53 0.83 0.43 0.28
**SF-BARI score** - 1 year - 2 years - 3 years - 4 years - 5 years	88.8 ± 21.0 91.1 ± 22.3 86.2 ± 24.2 83.8 ± 24.9 84.2 ± 24.9	86.5 ± 20.9 90.7 ± 22.7 82.7 ± 23.7 81.2 ± 21.9 79.1 ± 23.7	0.12 0.78 0.09 0.29 **0.046**

*RYGB* Roux-en-Y gastric bypass, *SG* sleeve gastrectomy, *BMI* body mass index, *%TWL* total body weight loss, %*EBMIL* excess BMI loss

^*^Binge eating disorder, sweet eating habit and night eating syndrome are summarized as “eating pattern”

Values are expressed as means ± standard deviation

Complications did not differ significantly between groups (CCI 2.1 ± 8.5 vs. 2.3 ± 7.4; *p* = 0.76). The full list of complications is provided in the supplementary material (Supp. Table 1 and Supp. Table 2).

### Binge Eating Disorder

Among patients diagnosed with BED, 228 (67.9%) underwent RYGB, while 108 (32.1%) underwent SG (Supp. Table 3). BMI was consistently lower at all time points in patients with BED who underwent RYGB than in patients who underwent SG. Similarly, %TWL was higher in the RYGB group at 1, 2, 3, and 5 years. %EBMIL was higher throughout all follow-up time points. The SF-BARI score showed better outcomes after RYGB at 2, 3, and 5 years.

### Sweet Eating Habit

A total of 288 patients (57.0%) underwent RYGB, and 112 (43.0%) underwent SG. BMI was consistently lower in patients who underwent RYGB at all time points. %TWL was significantly higher after RYGB at the 2-, 3-, and 4-year follow-ups, and %EBMIL was higher until the fourth postoperative year. The SF-BARI score was also higher in RYGB patients throughout the follow-up period, though the difference did not reach statistical significance in the fifth year. Detailed results are provided in Supp. Table 4.

### Night Eating Syndrome

Of the patients diagnosed with NES, 123 (64.1%) underwent RYGB, and 69 (35.9%) underwent SG. In contrast to BED and SEH, where RYGB led to higher %TWL and SF-BARI scores at most follow-up points, in patients with NES, these outcomes were only higher at 2 years (%TWL: 32.7 ± 8.4 vs. 29.2 ± 9.8, *p* = 0.03; SF-BARI score: 91.2 ± 22.4 vs. 78.8 ± 22.3, *p* < 0.01). Further details are presented in Supp. Table 5.

### Gender Differences

Female patients demonstrated a consistently higher %TWL at all follow-up time points, regardless of the presence of an EP. Among patients with an EP, the %TWL at 5 years was 29.5 ± 10.0 in females compared to 25.3 ± 9.9 in males (*p* < 0.01), and among those without an EP, the values were 28.9 ± 8.5 versus 24.3 ± 10.9 (*p* < 0.01), respectively. Additionally, female patients exhibited a higher SF-BARI score throughout the follow-up period. Follow-up rates were comparable between men and women.

### RYGB vs. SG

Most patients underwent RYGB (1083; 69.9%) with the majority being female (71.4%) (*p* < 0.01). Patients who underwent RYGB had significantly lower BMI than those who had SG (43.4 ± 5.3 kg/m^2^ vs. 44.1 ± 12.0 kg/m^2^; *p* < 0.01), and this difference persisted throughout all follow-up periods (*p* < 0.01). %TWL, %EBMIL as well as the SF-BARI score were higher in the RYGB throughout the 5-year follow-up period. Detailed values are presented in Table 3.

**Table 3:** Characteristics and outcomes after RYGB vs. SG

Characteristics	RYGB (*n* = 1083)	Sleeve (*n* = 467)	Total (*n* = 1550)	*p*-value
**Sex (f) (*n*; %)**	807; 74.5	300; 64.2	1107; 71.4	** < 0.01**
**Age at baseline (years)**	41.3 ± 12.6	44.1 ± 12.0	42.1 ± 12.5	** < 0.01**
**BMI (kg/m**^**2**^**)** - at baseline - 1 year - 2 years - 3 years - 4 years - 5 years	43.4 ± 5.3 29.5 ± 4.6 29.2 ± 4.9 30.0 ± 5.0 30.5 ± 5.2 30.9 ± 5.4	46.0 ± 7.9 31.8 ± 6.2 32.0 ± 6.1 32.9 ± 6.7 33.2 ± 6.4 33.0 ± 6.7	44.2 ± 6.3 30.2 ± 5.2 30.0 ± 5.5 30.9 ± 5.7 31.4 ± 5.7 31.6 ± 5.9	** < 0.01** ** < 0.01** ** < 0.01** ** < 0.01** ** < 0.01** ** < 0.01**
**%TWL** - 1 year - 2 years - 3 years - 4 years - 5 years	31.6 ± 7.2 32.6 ± 8.6 31.0 ± 9.1 29.8 ± 9.3 28.8 ± 9.9	30.4 ± 8.3 29.9 ± 9.6 27.7 ± 10.3 26.6 ± 9.6 26.9 ± 9.9	31.2 ± 7.5 31.7 ± 9.0 30.0 ± 9.6 28.9 ± 9.5 28.2 ± 9.9	**0.03** ** < 0.01** ** < 0.01** ** < 0.01** **0.04**
**%EBMIL** - 1 year - 2 years - 3 years - 4 years - 5 years	78.3 ± 21.1 80.0 ± 23.2 75.4 ± 24.1 72.3 ± 24.0 69.9 ± 25.4	71.0 ± 22.7 69.2 ± 24.3 64.8 ± 25.4 62.3 ± 23.2 63.6 ± 24.5	76.2 ± 21.8 76.7 ± 24.0 72.1 ± 25.0 69.3 ± 24.2 68.0 ± 25.3	** < 0.01** ** < 0.01** ** < 0.01** ** < 0.01** ** < 0.01**
**SF-BARI score** - 1 year - 2 years - 3 years - 4 years - 5 years	89.0 ± 20.2 92.6 ± 21.7 87.5 ± 23.8 86.0 ± 22.8 83.8 ± 24.0	84.4 ± 22.2 86.2 ± 23.5 79.0 ± 24.1 75.4 ± 24.4 78.4 ± 24.9	87.6 ± 20.9 90.6 ± 22.5 84.9 ± 24.1 82.8 ± 23.8 82.1 ± 24.3	** < 0.01** ** < 0.01** ** < 0.01** ** < 0.01** **0.02**

*RYGB* Roux-en-Y gastric bypass, *SG* sleeve gastrectomy, *BMI* body mass index, *TWL%* total body weight loss, *EBMIL%* excess BMI loss

Values are expressed as means ± standard deviation

## Discussion

The findings of this study provide invaluable insights into the relationship between preoperatively diagnosed EPs, including EDs, and postoperative outcomes in MBS. In our cohort, the majority of patients who underwent primary MBS had at least one diagnosed EP, with most showing features of multiple EPs. Despite the still existing common belief that EPs negatively affect long-term surgical success, our results suggest that preoperative EPs do not negatively impact weight loss or overall postoperative outcomes for up to 5 years. These findings challenge current guidelines that identify EDs as relative contraindications for MBS and highlight the need for a more nuanced approach to patient selection and postoperative care.

Below, we discuss the following observations: first, the impact of EPs on postoperative outcomes. Second, the gender differences in relation to the MBS outcomes, and third, its implications for surgical decision, comparing RYGB and SG.

### Impact of Eating Patterns on Postoperative Outcomes

Our data demonstrate that the majority (67.6%) of MBS patients exhibit some form of preoperative EP. According to Müller et al., BED is the most common EP and formally recognized ED in patients with obesity and those seeking surgical treatment in particular [28]. However, compared to prior studies, we did not only collect data on the most known EDs like BED but also on EPs like NES, SEH, FEH, SD, and EEH allowing a better representation of the MBS population.

Previous research by Cella et al. [29] and Ballardini et al. [30] confirm that patients with severe obesity who are candidates for MBS are frequently affected by EDs which can persist even after surgery. Athanasiadis et al. [13] identified sweet consumption, emotional eating, portion size, food urges, binge eating, and loss of control eating as risk factors for recurrent weight gain after MBS. This supports past and more recent studies that found that the presence of maladaptive eating behaviors, such as BED and emotional eating, is strongly correlated with poor postoperative weight loss [31].

Looking at our findings, while patients with an EP initially exhibited a higher %TWL within the first year postoperatively, this difference diminished over time, with no significant difference at 5 years between patients with an EP and without an EP. This suggests that while EP patients may initially respond more aggressively to bariatric interventions, long-term outcomes remain comparable across patient groups. However, clinically, a 1.4% difference in %TWL at 1 year is minimal and unlikely to affect patient outcomes meaningfully. These results contribute to a growing body of evidence suggesting that preoperative EPs, if well controlled, are not associated with poor long-term outcomes following MBS. Previous studies similarly found that preoperative BED does not impair postoperative weight loss [15, 16]. A recently published study of 426 patients examined the impact of preoperative eating habits on weight loss after MBS and, with a mean follow-up of 24 months, found no significant differences between patients with and without specific eating habits [32]. Our study confirms these results in a larger patient cohort with extended follow-up over 5 years. One potential explanation for our findings is the preoperative assessment by an experienced psychiatrist of all patients and excluding severe, uncontrolled EP in combination with other psychosocial issues and the improved psychological follow-up and nutritional counseling that patients with certain EPs may receive. This can lead to better long-term adherence to weight management strategies.

Importantly, after MBS, individuals who previously struggled with food addiction may experience a shift toward other disordered behaviors or addictions [33]. This phenomenon, often referred to as “addiction transfer” or “cross-addiction,” occurs because the underlying psychological and neurological factors driving addictive behaviors remain unresolved. For example, a person might replace compulsive eating with increased alcohol use, gambling, or shopping [34, 35]. Similarly, some may develop new patterns of substance abuse or engage in excessive exercise as a way to cope with emotional distress or fill the void left by food [36, 37]. This highlights the importance of comprehensive psychological support and monitoring in the postoperative period to address emerging maladaptive behaviors and ensure sustainable recovery.

Within our obesity center, psychological consultations are taken very seriously, and severe cases are discussed at our interdisciplinary board. Postoperative psychological support is always offered when needed, ensuring patients receive the necessary care to manage potential shifts in behavior. Patients who were not considered psychologically fit were not operated on. As a result, they were not included in this study, which represents a certain intentional selection bias. Targeted psychological interventions for BED patients have been shown to enhance postoperative weight loss [38]. Similarly, cognitive behavioral therapy interventions have demonstrated effectiveness in reducing eating pathology before MBS, leading to improved postoperative outcomes [39].

### Gender Differences in MBS Outcomes

A notable observation in our study is the superior outcomes observed in female patients compared to their male counterparts, irrespective of EP status. Although, female patients suffered more often from EPs with SEH and SB predominantly than males, female patients achieved significantly greater %TWL and higher SF-BARI scores at all times. Potential explanations for these differences include hormonal influences, greater adherence to postoperative lifestyle modifications, and societal impact regarding body image [40]. A German study showed that despite the higher incidence of obesity among men, women are significantly overrepresented in bariatric care proposing that reasons for this are likely multifaceted [41]. They observed that women are presumably subject to greater social pressure to conform to aesthetic norms resulting in obese women experiencing greater psychological distress than men with the same BMI, which may contribute to a higher motivation for therapy [41]. Moreover, women generally have a stronger awareness of health-related issues and preventive care [42]. Additionally, differences in visceral fat distribution contribute to earlier and more frequent metabolic comorbidities in men, potentially leading to worse postoperative outcomes [43]. These findings align with literature suggesting that weight loss after MBS is influenced by various clinical factors, including initial BMI, age, gender, and ethnicity [44]. While not all of these factors were directly investigated in this study, they warrant further exploration in future research to better tailor surgical gender-related recommendations and postoperative care strategies.

### Implications for Surgical Choice: RYGB vs. SG

Our study supports the notion that RYGB offers superior postoperative outcomes compared to SG, particularly in patients with certain EPs. RYGB was associated with a higher SF-BARI score and greater %TWL at all time points. This is consistent with previous studies that highlighted the long-term benefits of RYGB compared to SG [45, 46]. Notably, patients with BED and SEH demonstrated better outcomes with RYGB, whereas SG may be a more suitable option for individuals with EEH.

### Limitations

While our study offers valuable insights into preoperative EPs and their impact on postoperative outcomes in a large patient cohort, several limitations should be acknowledged. First, the lack of standardized diagnostic criteria for some EPs limits comparability across studies. Second, we were unable to assess postoperative disordered behaviors such as purging, excessive exercise, or laxative use, nor the possible shift to other addictive behaviors (e.g., substance use, gambling, or alcohol). Validated tools to predict post-surgical EDs are still in development, and no single measure is yet reliable for routine use. As a result, such tools were not systematically used preoperatively in this study. However, patients with apparent disordered eating received more intensive care and follow-up. Moreover, eating behaviors tend to evolve after surgery, but these changes were beyond the scope of our analysis. Additionally, follow-up rates for the SF-BARI score declined over time, from 74.0% at 1 year to 35.0% at 5 years. This was primarily due to missing diabetes status data and the requirement for five variables—weight loss, diabetes status, hypertension, dyslipidemia, and OSAS status—to calculate the score. However, our 5-year weight loss follow-up rate remained relatively high at 76.0%. Long-term follow-up in bariatric patients is a known challenge in the literature [47], highlighting the need for better strategies to improve adherence and ensure comprehensive outcome assessment. Another important limitation of our study is the inherent selection bias introduced by our comprehensive preoperative assessment process. Patients with severe, uncontrolled EPs and EDs were excluded from undergoing bariatric surgery after evaluation by our multidisciplinary team. As a result, our study population represents patients who were deemed suitable for surgery, potentially leading to an underestimation of the true prevalence and impact of EPs in the broader bariatric surgery candidate population. Finally, we lacked systematic data on the use of Obesity Management Medications (OMM). In our clinical practice, GLP-1 receptor agonists (RAs) were introduced specifically for the treatment of obesity starting in 2019, after the study period. Although some patients with T2D received GLP-1 RAs during the study period, their use was infrequent and typically at low doses unlikely to meaningfully affect weight loss outcomes. Moreover, pharmacotherapy specifically for obesity was not routinely implemented in our center at that time. Nonetheless, the absence of detailed OMM data limits our ability to fully assess the potential impact of adjunct pharmacotherapy on weight loss and related outcomes.

The strengths of this study lie in its large patient cohort, which included a wide range of EPs, and its long follow-up period of up to 5 years with a high follow-up rate for weight loss. Including %TWL, %EBMIL, and the use of composite endpoints further enhances the robustness of our findings. Additionally, as this study incorporates various types of EPs, the findings reflect a broader patient population.

## Conclusion

Our findings indicate that preoperatively diagnosed EPs do not negatively impact long-term outcomes in terms of weight loss and comorbidity resolution following MBS. These results call into question current guidelines, particularly the classification of severe EPs as contraindications for surgery, and highlight the potential benefits of personalized, procedure-specific surgical planning. Thorough preoperative assessment and postoperative follow-up are crucial to ensure optimal outcomes.

## Supplementary Information

Below is the link to the electronic supplementary material.

ESM 1 (DOCX 28.7 KB)

## Data Availability

No datasets were generated or analysed during the current study.
