# Supplementary material for: Preoperative Eating Patterns and Their Effect on Post-operative Outcomes in Metabolic and Bariatric Surgery: A Cohort Study of 1550 Patients
Source: Obes Surg. 2025 Aug 7;35(9):3717–28. doi: 10.1007/s11695-025-08094-y (PMC12457552; doi:10.1007/s11695-025-08094-y)
Supplement: Supplementary file 1 — (DOCX 28.7 KB) [file 11695_2025_8094_MOESM1_ESM.docx]

**Supplementary material**

Supp. Table 1 Complications of Patients with an Eating Pattern vs. with No Eating Pattern

| **Complications** | **Eating Pattern* (n=670)** | **No Eating Pattern (n=502)** | **p-value** |
| --- | --- | --- | --- |
| **Hematoma** | 1 (0.1%) | 0 (0.0%) | 1.00 |
| **Seroma** | 1 (0.1%) | 2 (0.4%) | 1.00 |
| **Wound infection** | 3 (0.3%) | 4 (0.8%) | 0.66 |
| **Bleeding** | 5 (0.7%) | 6 (1.2%) | 0.53 |
| **GI bleeding** | 2 (0.2%) | 2 (0.4%) | 1.00 |
| **Anastomotic leak** | 1 (0.1%) | 3 (0.6%) | 0.61 |
| **Intraabdominal**  **infection (including diagnostic laparoscopies)** | 2 (0.2%) | 5 (1.0%) | 0.15 |
| **Obstruction at the jejunojejunostomy** | 3 (0.3%) | 1 (0.2%) | 0.64 |
| **Trocar hernia** | 2 (0.2%) | 0 (0.0%) | 0.51 |
| **Stenosis** | 0 (0.0%) | 5 (1.0%) | 0.17 |
| **CCI** | 2.1 ± 8.5 | 2.3 ± 7.4 | 0.76 |

** Binge Eating, Sweet Eating, and Nightly Eating are summarized as “Eating Pattern”*

*GI=gastrointestinal; CCI=Comprehensive Complication Index*

Supp. Table 2 Complications of Patients requiring surgical revision (Clavien-Dindo ≥ 3b) with an Eating Pattern vs. with No Eating Pattern

|  | **Eating Pattern* (n=670)** | **No Eating Pattern (n=502)** |
| --- | --- | --- |
| **Overall** | n=15 | n=12 |
| **Complications**  **(Clavien-Dindo ≥ 3b)** | **Treatment** | **Treatment** |
| **Obstruction at the jejunojejunostomy** | - diagnostic laparoscopy, creation of two new small bowel side-to-side anastomoses (2x) (RYGB) - diagnostic laparoscopy,   placement of a gastrostomy catheter (2x) (RYGB)   - laparotomy and re-creation of the jejunojejunostomy (RYGB) | - diagnostic laparoscopy, creation of two new small bowel side-to-side anastomoses (RYGB). |
| **Bleeding** | - Postoperative bleeding at the staple line: diagnostic laparoscopy (SG). - Bleeding (RYGB) from the right epigastric artery: laparotomy (RYGB) | - Arterial staple‑line bleeding: laparoscopic hemostasis (RYGB). - Bleeding from short gastric vessels: laparoscopic hemostasis (SG). - Bleeding from drain tract: hemostasis via the drain channel and revisional laparoscopy (SG), |
| **Internal hernia/diagnostic laparoscopy** | - Diagnostic laparoscopy and closure of mesenteric defect (RYGB). - Unclear infection focus: diagnostic laparoscopy without findings (RYGB). | - Diagnostic laparoscopy and closure of mesenteric defect (RYGB). - Unexplained intra‑abdominal symptoms (suspected internal hernia): diagnostic laparoscopy (no abnormality found) (RYGB). |
| **Anastomotic leak/**  **Bowel leak** | - Microleak in an anisoperistaltic bypass reconstruction in the setting of incomplete intestinal malrotation and aspiration pneumonia: diagnostic laparoscopy converted to open adhesiolysis, partial omentectomy, completion gastrectomy, and segmental small bowel resection, followed by reconstruction with a Roux-en-Y end-to-side esophagojejunostomy and additional enteroenterostomies.(RYGB). | - Anastomotic leak after torsion of the jejuno‑jejunostomy with internal hernia : oversewing of the anastomosis, laparoscopic adhesiolysis and revision, conversion to biliopancreatic diversion (RYGB). - Gastroesophageal anastomotic insufficiency with iatrogenic injury to the common channel: laparotomy, resection of the pouch‑jejunal anastomosis, end‑to‑side esophago-jejunostomy, and reconstruction of the jejuno‑jejunostomy (RYGB). - Small‑bowel leak at the alimentary limb: revision with oversewing (RYGB). |
| **Anastomotic ulcer** | - | - Persistent anastomotic ulcer: gastric resection with creation of an esophagojejunostomy (RYGB). |
| **Trocar hernia** | - Suspected trocar hernia: abdominal wall revision – hernia not confirmed (RYGB). - Incarcerated trocar hernia: Fascial plication using the Mayo technique (RYGB). | - |
| **Wound infection** | - Wound infection: VAC therapy (RYGB). - Wound healing disorder: wound debridement and application of a VAC dressing (SG). - Abdominal wall revision with evacuation of seroma and drainage (RYGB). | - |
| **Pouch revision** | - | - Pouch necrosis: resection of the pouch (RYGB). |
| **Other** | - | - Minor liver laceration with bile leack: revisional laparoscopy with lavage and drainage (RYGB). |

** Binge Eating, Sweet Eating, and Nightly Eating are summarized as “Eating Pattern”*

*RYGB=Roux-en-Y gastric bypass; SG=sleeve gastrectomy; VAC=vacuum assisted closure-therapy*

Supp. Table 3 Outcomes of patients diagnosed with Binge Eating Disorder

| **Characteristics** | **RYGB (n=228)** | **SG (n=108)** | **p-value** |
| --- | --- | --- | --- |
| **BMI (kg/m^2^)**  - at baseline  - 1 year  - 2 years  - 3 years  - 4 years  - 5 years | 44.0 ± 5.5  29.5 ± 4.8  29.3 ± 5.4  30.3 ± 5.5  30.5 ± 5.5  30.6 ± 5.3 | 46.7 ± 8.2  32.8 ± 5.9  33.7 ± 6.1  33.9 ± 7.0  33.7 ± 6.9  34.3 ± 7.1 | **<0.01**  **<0.01**  **<0.01**  **<0.01**  **<0.01**  **<0.01** |
| **%TWL**  - 1 year  - 2 years  - 3 years  - 4 years  - 5 years | 32.7 ± 7.0  33.4 ± 9.0  31.5 ± 9.6  30.1 ± 10.4  29.5 ± 10.1 | 29.9 ± 8.7  27.8 ± 9.5  26.6 ± 10.0  26.9 ± 9.7  24.4 ± 10.4 | **<0.01**  **<0.01**  **<0.01**  0.10  **<0.01** |
| **%EBMIL**  - 1 year  - 2 years  - 3 years  - 4 years  - 5 years | 79.3 ± 21.2  80.3 ± 24.0  75.1 ± 24.6  72.6 ± 26.3  71.4 ± 25.3 | 67.1 ± 21.3  62.6 ± 23.6  61.6 ± 26.9  62.2 ± 24.9  57.1 ± 26.4 | **<0.01**  **<0.01**  **<0.01**  **0.03**  **<0.01** |
| **SF-Bari Score**  - 1 year  - 2 years  - 3 years  - 4 years  - 5 years | 83.6 ± 18.8  90.7 ± 23.1  86.5 ± 24.8  82.0 ± 23.5  82.9 ± 23.8 | 79.3 ± 21.1  80.7 ± 23.0  76.2 ± 24.8  74.5 ± 27.6  73.3 ± 29.6 | 0.10  **<0.01**  **0.01**  0.11  **0.04** |

*Values are expressed as means ± standard deviation. RYGB: Roux-en-Y gastric bypass; SG: Sleeve gastrectomy; BMI: Body Mass Index; %TWL: Total Body Weight Loss; %EBMIL: Excess BMI Loss*

Supp. Table 4 Outcomes of patients diagnosed with Sweet Eating Habit

| **Characteristics** | **RYGB (n=288)** | **SG (n=112)** | **p-value** |
| --- | --- | --- | --- |
| **BMI (kg/m^2^)**  - at baseline  - 1 year  - 2 years  - 3 years  - 4 years  - 5 years | 43.5 ± 5.3  29.3 ± 4.5  28.6 ± 4.7  29.4 ± 4.9  29.9 ± 5.1  29.8 ± 4.8 | 46.3 ± 8.3  31.4 ± 5.9  31.8 ± 5.7  32.7 ± 6.5  33.8 ± 7.1  32.2 ± 7.1 | **<0.01**  **<0.01**  **<0.01**  **<0.01**  **<0.01**  **0.01** |
| **%TWL**  - 1 year  - 2 years  - 3 years  - 4 years  - 5 years | 32.6 ± 7.4  33.9 ± 8.9  32.0 ± 8.9  30.6 ± 10.0  30.1 ± 9.8 | 31.1 ± 8.9  30.3 ± 9.9  28.7 ± 10.4  27.1 ± 10.5  27.5 ± 11.5 | 0.12  **<0.01**  **0.03**  **0.06**  0.16 |
| **%EBMIL**  - 1 year  - 2 years  - 3 years  - 4 years  - 5 years | 79.5 ± 19.5  82.5 ± 22.2  77.8 ± 22.3  74.6 ± 24.1  74.0 ± 23.9 | 72.7 ± 23.0  69.7 ± 24.3  66.5 ± 26.9  62.2 ± 25.2  66.4 ± 29.2 | **0.01**  **<0.01**  **<0.01**  **<0.01**  0.08 |
| **SF-Bari Score**  - 1 year  - 2 years  - 3 years  - 4 years  - 5 years | 85.2 ± 19.9  92.1 ± 22.5  88.4 ± 23.4  83.8 ± 23.8  85.4 ± 25.4 | 80.3 ± 21.0  83.1 ± 23.3  78.9 ± 23.6  72.7 ± 27.1  77.7 ± 28.5 | **0.06**  **<0.01**  **0.01**  **0.01**  0.09 |

*Values are expressed as means ± standard deviation. RYGB: Roux-en-Y gastric bypass; SG: Sleeve gastrectomy; BMI: Body Mass Index; %TWL: Total Body Weight Loss; %EBMIL: Excess BMI Loss*

Supp. Table 5 Outcomes of patients diagnosed with Night Eating Syndrome

| **Characteristics** | **RYGB (n=123)** | **SG (n=69)** | **p-value** |
| --- | --- | --- | --- |
| **BMI (kg/m^2^)**  - at baseline  - 1 year  - 2 years  - 3 years  - 4 years  - 5 years | 43.2 ± 5.4  29.5 ± 4.7  28.6 ± 4.6  29.7 ± 4.9  30.0 ± 4.9  30.2 ± 5.0 | 47.1 ± 9.0  32.5 ± 7.0  33.1 ± 6.4  32.8 ± 7.4  31.2 ± 5.2  34.3 ± 6.3 | **<0.01**  **<0.01**  **<0.01**  **0.03**  0.39  **<0.01** |
| **%TWL**  - 1 year  - 2 years  - 3 years  - 4 years  - 5 years | 31.7 ± 7.1  32.7 ± 8.4  30.6 ± 9.9  29.7 ± 9.7  28.5 ± 10.2 | 31.3 ± 8.9  29.2 ± 9.8  27.9 ± 11.4  27.4 ± 11.3  28.5 ± 9.3 | 0.76  **0.03**  0.26  0.40  0.99 |
| **%EBMIL**  - 1 year  - 2 years  - 3 years  - 4 years  - 5 years | 78.9 ± 21.4  81.9 ± 22.2  75.8 ± 24.4  73.8 ± 24.4  71.9 ± 26.0 | 70.9 ± 23.6  65.8 ± 24.0  66.7 ± 30.2  67.0 ± 25.4  61.8 ± 19.9 | **0.04**  **<0.01**  0.14  0.32  0.14 |
| **SF-Bari Score**  - 1 year  - 2 years  - 3 years  - 4 years  - 5 years | 83.7 ± 18.6  91.2 ± 22.4  88.6 ± 25.0  84.1 ± 24.4  80.5 ± 22.1 | 79.6 ± 20.9  78.8 ± 22.3  78.7 ± 31.1  71.6 ± 27.2  77.8 ± 22.0 | 0.22  **<0.01**  0.11  0.07  0.65 |

*Values are expressed as means ± standard deviation. RYGB: Roux-en-Y gastric bypass; SG: Sleeve gastrectomy; BMI: Body Mass Index; %TWL: Total Body Weight Loss; %EBMIL: Excess BMI Loss*
